# Supplementary material for: Feeling the Heat: The Campylobacter jejuni HrcA Transcriptional Repressor Is an Intrinsic Protein Thermosensor
Source: Biomolecules. 2021 Sep 27;11(10):1413. doi: 10.3390/biom11101413 (PMC8533110; doi:10.3390/biom11101413)
Supplement: Supplementary file 1 [file biomolecules-11-01413-s001.zip › biomolecules-1357203-supplementary.pdf]

Supplementary Materials

Feeling the heat: the *Campylobacter jejuni* HrcA transcriptional repressor is an intrinsic protein thermosensor

Giovanni Versace, Marta Palombo, Anna Menon, Vincenzo Scarlato * and Davide Roncarati *

Table of contents Page

Figure S1: HspR binding in vitro to P*cbp* promoter is unaffected up to 45°C 2
Figure S2: EMSA assay of HrcA binding to P*gro* in the presence or absence of GroE 2
Figure S3. EMSA assay of HrcA and HspR simultaneous binding to P*gro* at different temperatures 3
Figure S4. DNA-binding experiments of HrcA exposed to different temperatures and recovered at 25°C in the absence or presence of the GroE chaperonin 4
Table S1: List of strains and plasmids used in this study 5
Table S2: List of oligonucleotides used in this study 6

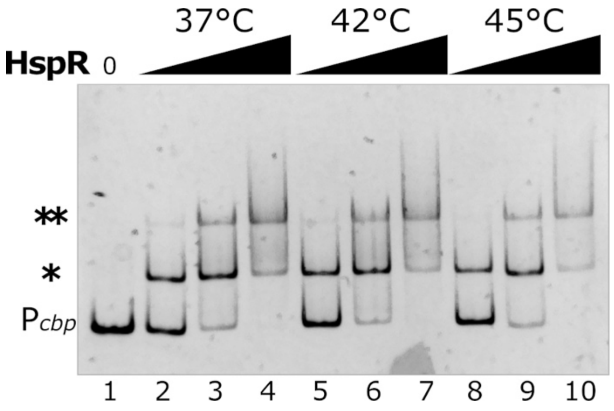

Figure S1. HspR binding in vitro to P*cbp* promoter is unaffected even at 45°C. EMSA carried out with purified HspR on P*cbp* promoter probe. DNA-protein complexes were allowed to form for 10 min at 25°C, then reactions were moved to different temperatures (37, 42 or 45°C) for 10 min, before loading the reactions on a native polyacrylamide gel. On the left, asterisks mark the different HspR shifted bands, while the label "P*cbp*" indicates the free probe. Lanes 1 to 4 contain 0, 15, 30 and 60 nM HspR, respectively. Lanes 4 to 6 and 8 to 10 contain the same increasing concentrations of HspR as in samples 2 to 4. Symbols are as in Figure 2. Symbols * and ** mark HspR shifted bands.

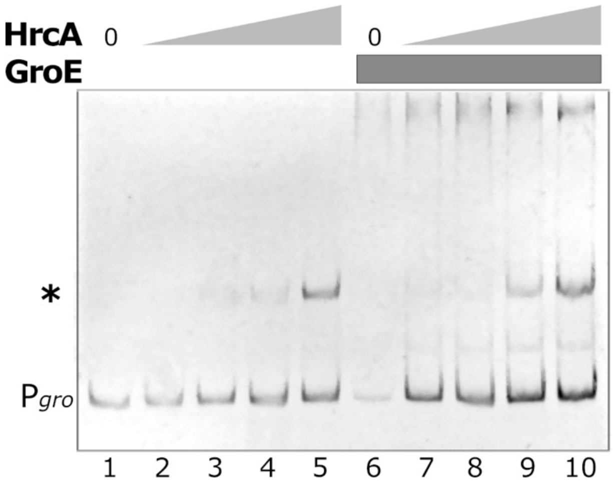

Figure S2. EMSA assay of HrcA binding to P*gro* in the presence or absence of GroE. EMSA carried out with purified HrcA on P*gro* promoter probe. Protein-DNA mixes were incubated for 30 min at 25°C in the presence of a 1.5-fold molar excess of GroES/GroEL (lanes 6 to 10) or GST control, (lanes 1 to 5) with respect to the highest HrcA concentration, before loading the reactions on a native polyacrylamide gel for electrophoretic separation. On the left, an asterisk marks the single HrcA shifted band, while the label "P*gro*" indicates the free probe. Lanes 1 to 5 contain 0, 18.7, 37.5, 75 and 150 nM HrcA, respectively. Lanes 6 to 10 contain the same increasing concentrations of HrcA as in samples 1 to 5. Symbols are as in Figure 6. The asterisk * indicates the HrcA shifted band.

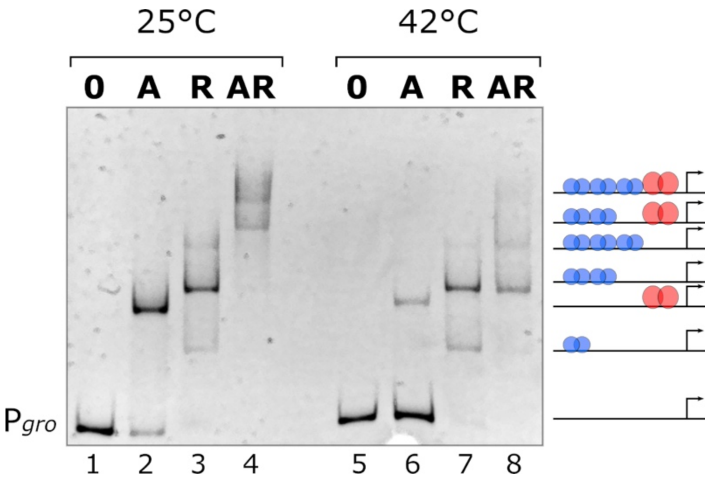

Figure S3. EMSA assay of HrcA and HspR simultaneous binding to P*gro* at different temperatures. EMSA carried out with purified HspR and HrcA proteins on P*gro* promoter probe. Protein-DNA mixes were incubated for 10 min at 25°C, then moved to the indicated temperatures for 10 min, before loading the reactions on a native polyacrylamide gel for electrophoretic separation. On the right, schematic representation of the putative complexes generating the shifted bands visible in the gel: red and blue ovals represent the HrcA and HspR proteins, respectively, the black horizontal line depicts the DNA probe. The bent arrow marks the position of the transcriptional start site. Symbols on the top of the gel: 0: no protein; A: HrcA only (360 nM); R: HspR only (60 nM); AR: HrcA and HspR (360 nM and 60 nM, respectively).

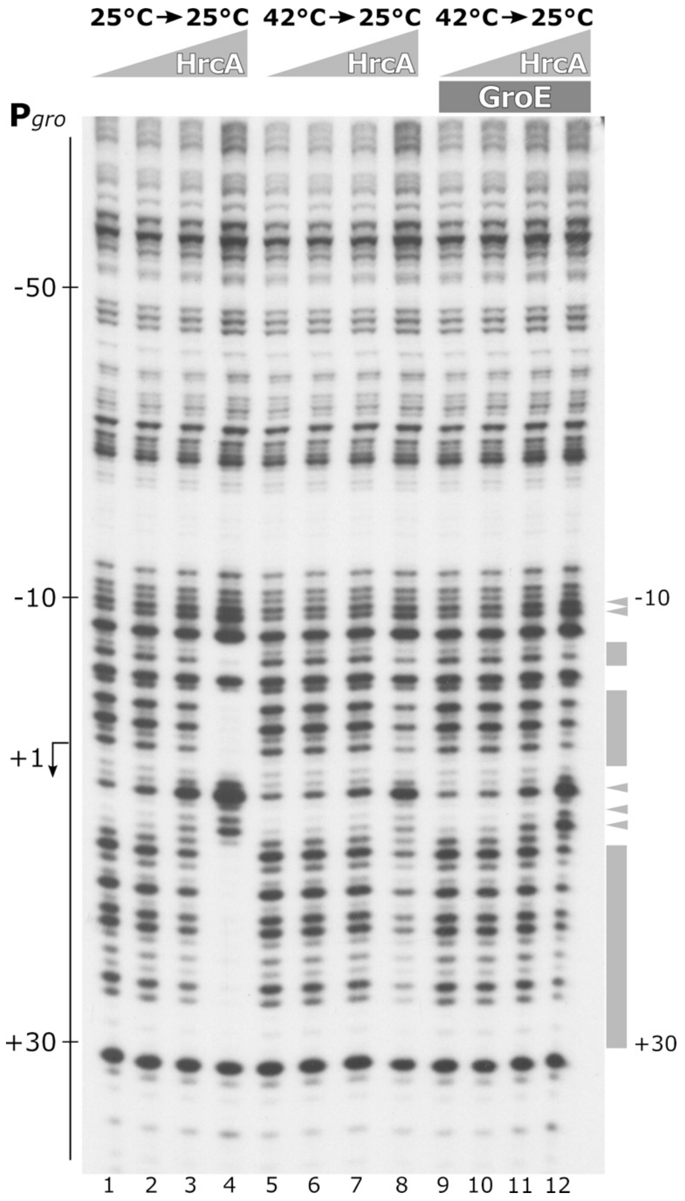

Figure S4. DNA-binding experiments of HrcA exposed to different temperatures and recovered at 25°C in the absence or presence of the GroE chaperonin. DNase I footprinting assay with HrcA on P*gro* labelled probe. Protein-DNA mixes were incubated for 10 min at 25°C, then moved to 25 or 42°C for 10 min, followed by a recovery step at permissive (25°C) temperature, in the absence or presence of a two-fold molar excess of GroESL (lanes 9 to 12) with respect to the highest HrcA concentration of the purified GroE chaperonin, before DNase I cleavage. Lanes 1 to 4, 5 to 8 and 9 to 12 contain 0, 45, 90 and 180 nM HrcA, respectively. Part of the experiment included in this figure (lanes 1 to 8) is the same presented in Figure 5B. On the left of the autoradiograph, the numbers refer to the positions with respect to the transcriptional start site (indicated by a bent arrow). Protected regions and DNase I hypersensitive sites are indicated on the right by grey boxes and arrowheads, respectively, together with positions delimiting the HrcA binding site.

Table S1 - List of strains and plasmids used in this study

Strain	Description	Reference
E. coli DH5α	*supE44 ΔlacU169 (ϕ80 lacZΔM15) hsdR17 recA1 endA1 gyrA96 thi-1 relA1*	[3]
E. coli BL21(DE3)	*hsdS gal (λcIts857 ind1 Sam7 nin5 lacUV5-T7 gene 1)*	[4]
C. jejuni NCTC11168	Wild type strain	[1]
**Plasmid**	**Description**	
pGEM-T-Easy	Cloning vector; Amp^R	Promega
pGEM-T-Easy-CjP*gro*	pGEM-T-Easy derivative, carrying a 261 bp PCR fragment (amplified with primers CjPgro-F and CjPgro-R) encompassing the P*gro* promoter.	[2]
pGEM-T-Easy-CjP*cbp*	pGEM-T-Easy derivative, containing a 285 bp PCR fragment (amplified with primers CjPcbp-F and CjPcbp-R) encompassing the *htrA-cbpA* intergenic region and the 5′ ends of the two genes.	[2]
pET15b	Expression vector, it allows N-terminal 6X-histidine-tag gene fusion; Amp^R	Novagen
pET15b-*hrcA*	pET15b derivative, containing the *hrcA* coding sequence amplified by PCR from chromosomal DNA of *C. jejuni* NCTC11168, digested with restriction enzymes NdeI and XhoI and ligated to pET15b.	[2]
pET15b-*hspR*	pET15b derivative, containing the *hspR* coding sequence amplified by PCR from chromosomal DNA of *C. jejuni* NCTC11168, digested with restriction enzymes NdeI and XhoI and ligated to pET15b.	[2]
pET15b-*groES*	pET15b derivative, containing the *groES* coding sequence amplified by PCR with primers CjGroES-F and CjGroES-R from *C. jejuni* NCTC11168 chromosomal DNA and cloned NdeI and XhoI into pET15b.	This work
pET15b-*groEL*	pET15b derivative, containing the *groEL* coding sequence amplified by PCR with primers CjGroEL-F and CjGroEL-R from *C. jejuni* NCTC11168 chromosomal DNA and cloned NdeI and XhoI into pET15b.	This work
$pGEX_{NN}$	Expression vector, it allows N-terminal GST gene fusion; Amp^R.	Novagen
$pGEX_{NN}$-*hrcA*	$pGEX_{NN}$ derivative, containing the *hrcA* coding sequence excised from pET15b-*hrcA* by NdeI/XhoI digestion.	[2]

Table S2 - List of oligonucleotides used in this study

Oligonucleotide	Sequence (5' to 3')[a]	RS
CjPgro-F	GGATCCGCACAACAACAAAAGCTACAATGCC	BamHI
CjPgro-R	CTCGAGCGCGTTTAACTAGAACACGCTTTCCTAAAGG	XhoI
CjPcbp-F	GGATCCCTTGCAGCAAATAAAGCACTTGCTAAAC	BamHI
CjPcbp-R	CTCGAGGCTAACTCCAAGAGTTTCGTATAAACTATTC	XhoI
CjGroES-F	ATATCATATGAATTTTCAACCTTTAGGAAAG	NdeI
CjGroES-R	ATATCTCGAGTTATTTTAAAATTCCTAAGATATCATC	XhoI
CjGroEL-F	ATATCATATGGCAAAAGAAATTATTTTTTCAG	NdeI
CjGroEL-R	ATATCTCGAGTTACATCATTCCGCCCATG	XhoI

[a] Restriction sites added for cloning purposes are underlined.
RS: restriction recognition site

References

1. Parkhill, J.; Wren, B.W.; Mungall, K.; Ketley, J.M.; Churcher, C.; Basham, D.; Chillingworth, T.; Davies, R.M.; Feltwell, T.; Holroyd, S.; Jagels, K.; Karlyshev, A.V.; Moule, S.; Pallen, M.J.; Penn, C.W.; Quail, M.A.; Rajandream, M.A.; Rutherford, K.M.; van Vliet, A.H.; Whitehead, S.; Barrell, B.G. The genome sequence of the food-borne pathogen Campylobacter jejuni reveals hypervariable sequences. *Nature* **2000**, *403*, 665–668, doi:10.1038/35001088.
2. Palombo, M.; Scarlato, V.; Roncarati, D. Cooperative regulation of Campylobacter jejuni heat-shock genes by HspR and HrcA. *Microorganisms* **2020**, *8*, 1161, doi:10.3390/microorganisms8081161.
3. Hanahan, D. Studies on transformation of Escherichia coli with plasmids. *J. Mol. Biol.* **1983**, *166*, 557–580, doi:10.1016/s0022-283680284-8.
4. Studier, F.W.; Rosenberg, A.H.; Dunn, J.J.; Dubendorff, J.W. Use of T7 RNA polymerase to direct expression of cloned genes. *Methods Enzymol.* **1990**, *185*, 60–89, doi:10.1016/0076-687985008-c.
